# Association of Toll-Like Receptor 4 Polymorphisms with Diabetic Foot Ulcers and Application of Artificial Neural Network in DFU Risk Assessment in Type 2 Diabetes Patients

**DOI:** 10.1155/2013/318686

**Published:** 2013-07-11

**Authors:** Kanhaiya Singh, Vivek Kumar Singh, Neeraj K. Agrawal, Sanjeev K. Gupta, Kiran Singh

**Affiliations:** ^1^Department of Molecular & Human Genetics, Banaras Hindu University, Varanasi 221005, India; ^2^Department of Mining Engineering, Indian Institute of Technology, Banaras Hindu University, Varanasi 221005, India; ^3^Department of Endocrinology and Metabolism, Institute of Medical Sciences, Banaras Hindu University, Varanasi 221005, India; ^4^Department of General Surgery, Institute of Medical Sciences, Banaras Hindu University, Varanasi 221005, India

## Abstract

The Toll-Like receptor 4 (TLR4) plays an important role in immunity, tissue repair, and regeneration. The objective of the present work was to evaluate the association of TLR4 single nucleotide polymorphisms (SNPs) rs4986790, rs4986791, rs11536858 (merged into rs10759931), rs1927911, and rs1927914 with increased diabetic foot ulcer (DFU) risk in patients with type 2 diabetes mellitus (T2DM). PCR-RFLP was used for genotyping TLR4 SNPs in 125 T2DM patients with DFU and 130 controls. The haplotypes and linkage disequilibrium between the SNPs were determined using Haploview software. Multivariate linear regression (MLR) and artificial neural network (ANN) modeling was done to observe their predictability for the risk of DFU in T2DM patients. Risk genotypes of all SNPs except rs1927914 were significantly associated with DFU. Haplotype ACATC (*P* value = 9.3*E* − 5) showed strong association with DFU risk. Two haplotypes ATATC (*P* value = 0.0119) and ATGTT (*P* value = 0.0087) were found to be protective against DFU. In conclusion TLR4 SNPs and their haplotypes may increase the risk of impairment of wound healing in T2DM patients. ANN model (83%) is found to be better than the MLR model (76%) and can be used as a tool for the DFU risk assessment in T2DM patients.

## 1. Introduction

The prevalence of type 2 diabetes and its progression in the present world is rising at an alarming rate with 330 million of worldwide population likely to be affected by this metabolic disorder in the coming decade [1]. Impairment in wound healing is a serious complication of type 2 diabetes and it withholds a huge percentage of all the amputations performed worldwide [2]. It has been estimated that up to 25% of T2DM patients may develop DFU once in their life time [3]. A normal wound healing process progresses through a short inflammatory phase via proliferative phase to remodeling phase which are required to provide sufficient wound strength and closure of wound in an appropriate time [4]. Any imbalance in these phases will lead to a condition when wounds are not able to heal properly and will result into chronic wounds. Type 2 diabetes results in decrease of growth factors and cytokines required for the proper proliferative phase, increase in proinflammatory cytokines which result in spread of inflammatory phase, and increase in matrix degrading enzymes which disrupt the remodeling phase by degrading newly synthesized matrix and hence together bring impairment in wound healing [5, 6]. 

For proper wound healing the inflammation phase should be well coordinated and should not spill into proliferation and remodeling phase. Infection status of the wound also accounts for an important variable which decides the fate of the wound. Prolonged inflammation and infection will lead to the formation of chronic wound which either takes long time to heal or does not heal at all [7]. Toll-like receptors (TLRs) in mammals are homologous to Toll receptors discovered in *Drosophila* and are known to mediate innate immunity by producing antimicrobial peptides along with various chemokines and cytokines. TLR4 is one of the most extensively studied members of TLR family and is shown to be a key effector of the immune system by recognizing PAMPs (pathogen associated molecular patterns) over bacteria and viruses [8, 9]. TLR4 plays an important role in wound healing [10] and any sort of imbalance in TLR4 mediated signaling may abrogate the proper wound healing cascade [11, 12]. Ruzehaji et al. have shown that a cytoskeletal protein Flightless I modulate wound inflammation, angiogenesis, and remodeling which act via TLR4-MyD88 signaling pathway [11]. Our group has recently shown that differential expression of TLR4 in human diabetic wounds leads to impairment in wound healing cascade and finally into chronic nonhealing ulcers [12]. Deregulation of the TLR4 signaling due to the single nucleotide polymorphisms (SNPs) in the extracellular domain of TLR4 may alter the ligand binding capacity [13] and disturbs the pro- and anti-inflammatory cytokines, hence modulating the risk of chronic inflammation, thereby delaying wound healing. Recently two cosegregating SNPs which result in the change of amino acids in the extracellular domain of TLR4 have been identified [13]. These SNPs, namely, Asp299Gly (rs4986790) and Thr399Ile (rs4986791), affect the TLR4 mediated effector functions in a variety of ways. Apetoh et al. [14] reported that these polymorphisms reduce the binding efficiency of TLR4 with its endogenous and exogenous ligands, while Prohinar et al. [15] reported that these polymorphisms reduce the extracellular accumulation of functional TLR4 thereby resulting in inadequate TLR4 signaling in response to microbial infection. Three more SNPs, namely, rs11536858 (now merged into rs10759931), rs1927911, and rs1927914 of TLR4 gene are also reported to be associated with inflammatory diseases including cancer [16]. Therefore, in the present study we aimed to find diabetic foot ulcer (DFU) risk associated with rs4986790, rs4986791, rs11536858, rs1927911, and rs1927914 in the TLR4 gene in type 2 diabetes mellitus (T2DM) patients. Multiple linear regression (MLR) and artificial neural network (ANN) modeling were used for the assessment of these SNPs as a risk factor for DFU in T2DM patients. 

## 2. Materials and Methods

### 2.1. Sample Collection

A total of 255 individuals including 125 T2DM patients with DFU and 130 age matched controls were enrolled for this hospital based case control study. Recruitment of patients was done from OPD clinics of the University Hospital, Institute of Medical Sciences, Banaras Hindu University, Varanasi, India. All the cases were subjected for clinical and laboratory evaluation (Table 1) and the family history, habits, and duration of disease were recorded through a questionnaire. A total of 130 non-type 2 diabetic individuals belonging to similar ethnicity, with controlled fasting or postprandial sugar levels and without any other inflammatory or chronic disease, were included in the study as controls. Informed consent was obtained from all the subjects to carry out molecular analysis. Institutional ethical committee approval was obtained. 

### 2.2. Genotyping of SNPs by PCR-RFLP

**Figure 1:**
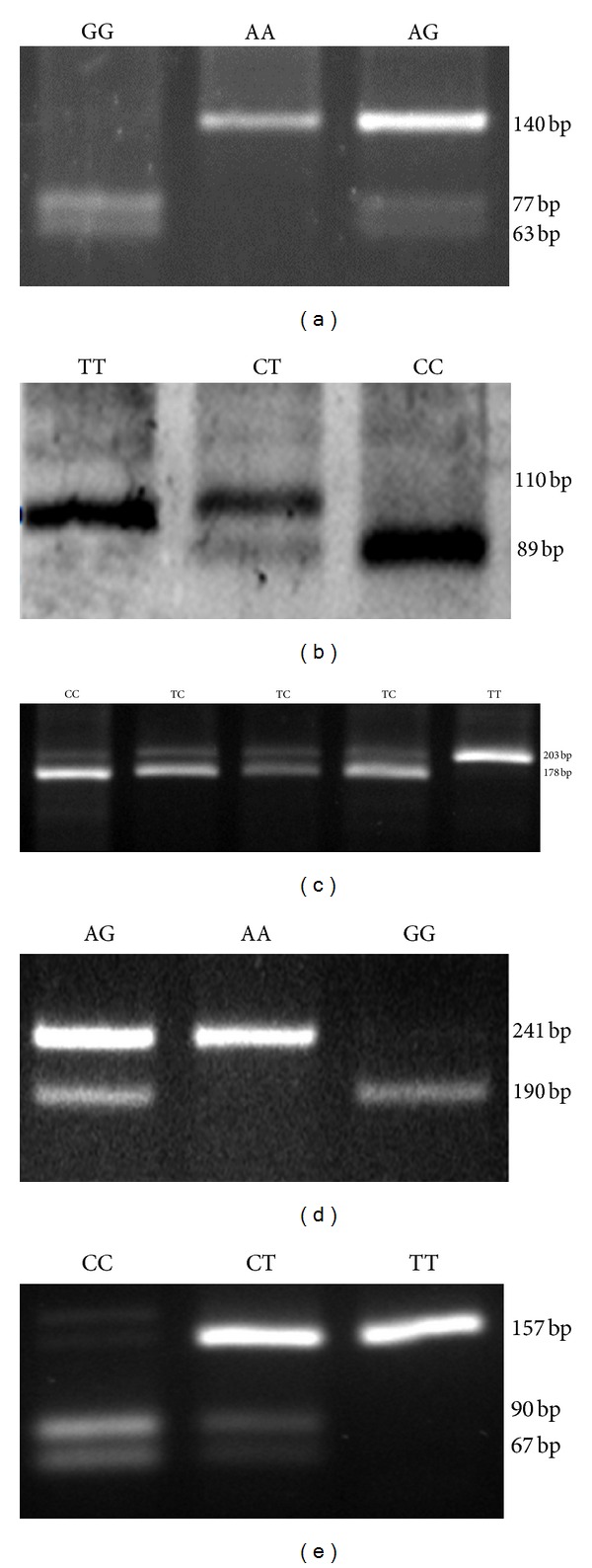
Polymerase chain reaction-restriction fragment length polymorphism (PCR-RFLP) analysis of five SNPs of the Toll-like receptor 4 (TLR4) gene. (a) For genotyping rs4986790, the 140 bp PCR product was digested with *BccI*. The A allele is not cut by the enzyme, whereas the G allele yields 77 and 63 bp products. (b) For genotyping rs4986791, the 110 bp PCR product was digested with *BslI*. The T allele is not cut by the enzyme, whereas the C allele yields 89 and 21 bp products. (c) For genotyping rs1927911, the 203 bp PCR product was digested with *StyI*. The T allele is not cut by the enzyme, whereas the C allele yields 178 and 25 bp products. (d) For genotyping rs11536858, the 241 bp PCR product was digested with *KpnI*. The A allele is not cut by the enzyme, whereas the G allele yields 190 and 51 bp products. (e) For genotyping rs1927914, the 157 bp PCR product was digested with *SphI*. The T allele is not cut by the enzyme, whereas the C allele yields 90 and 67 bp products.

Genomic DNA was extracted from peripheral blood using standard salting-out procedure. The SNPs of TLR4 gene, namely, Asp299Gly (rs4986790), Thr399Ile (rs4986791), rs11536858, rs1927911, and rs1927914 were analyzed using polymerase chain reaction restriction fragment length polymorphism (PCR-RFLP). The methodology and the implicated primers in the study are provided in Table 2. The PCR reaction set up was composed of an initial denaturation step of 5 minutes followed by 35 cycles of 40 seconds at 94°C, 45 seconds 58°C, and 40 seconds at 72°C. It was then followed by a final extension step of 10 minutes. The amplified products of the SNPs rs4986790, rs4986791, rs11536858, rs1927911, and rs1927914, were digested with restriction enzymes *BccI*, *BslI*, *KpnI*, *StyI*,* and SphI*, respectively. The restricted products were separated on 3% agarose gel (Figure 1).

### 2.3. Statistical Analysis for Genotype Comparison

Allele and genotype distribution among groups were evaluated using chi-square test. The difference in the frequencies between the case and the control groups was analyzed for statistical significance at the 95% confidence interval (CI) using *χ* ^2^ test. The allele frequencies of all SNPs were in Hardy-Weinberg equilibrium. Odds ratios (ORs) were calculated and reported within the 95% confidence limits. A two-tailed *P* value of ≤0.05 was considered as statistically significant. Power (sensitivity) of the study was calculated using software GraphPad Prism. Power > 80% is considered as statistically significant. 

### 2.4. Linkage Disequilibrium (LD) and Haplotype Analysis

Haplotype frequencies and LD were calculated using Haploview software (Version 4.2) developed at “The Broad Institute” (http://www.broadinstitute.org/), which is based on the EM algorithm. The standardized disequilibrium coefficient (*D*′) and correlation coefficient (*r* ^2^) between these SNPs were also analyzed using the LD plot function of this software to find certain allelic combinations of SNPs above that may alter the risk of DFU. 

### 2.5. ANN Modelingfor DFU Risk Assessment

Artificial neural networks (ANN) have emerged as a result of simulation of biological nervous system, such as the brain on a computer. Artificial neural networks are represented as a set of nodes called neurons and connections between them. The connections have weights associated with them, representing the strength of those connections. Nowadays neural networks can be applied to problems that do not have algorithmic solutions or problems for which algorithmic solutions are too complex to be found. In other words the kind of problems in which inputs and outputs variables do not have a clear relationship between them, a neural network is an efficient approach in such problems. Several research groups have suggested ANN as a useful approach for genetic epidemiology. We have tried to model complex relationship between the genotypes and occurrence of DFU.

The neural network paradigm adopted in this study utilizes the back-propagation learning algorithm [17]. A standard back propagation neural network consists of a number of interconnected processing units, commonly referred to as “artificial neurons.” Neurons are arranged into two or more layers and interact with each other via weighted connections. These scalar weights determine the nature and strength of influence between the interconnected neurons. Each neuron is connected to all the neurons in the next layer. There is an input layer where data are presented to the neural network, and an output layer that holds the response of the network to the input. It is the intermediate layers, known as hidden layers that enable these networks to represent and compute complicated associations between patterns. The network once trained allows complex problems to be solved without requiring the detailed dynamics of the actual system. 

The back-propagation training algorithm is an iterative gradient process designed to minimize the mean square error between the actual output of a multilayer feedforward network and the desired output. Network is trained using a simple teacher-enforced training. The back-propagation training algorithm based on Levenberg-Marquardt back propagation. LM was used to train the present model. Levenberg-Marquardt back propagation is a fast back propagation algorithm and is recommended as a first choice for supervised learning.

 First, difference between the desired output and the network outputs, that is, errors, for all the training patterns are calculated. The weights (*w*) are updated using the Hessian matrix as defined in the following:

(1) $$\begin{array}{c} \Delta w=-H^{-1}g,\\ w_{k+1}=w_{k}+\Delta w, \end{array}$$

where

(2) $$\begin{array}{c} H=\left[\begin{array}{cccc} \frac{\delta^{2}E}{\delta w_{1}^{2}} & \frac{\delta^{2}E\;}{\delta w_{1}\delta w_{2}} & \cdots & \frac{\delta^{2}E}{\delta w_{1}\delta w_{n}}\\ \frac{\delta^{2}E}{\delta w_{2}\delta w_{1}} & \frac{\delta^{2}E}{\delta w_{2}^{2}} & \cdots & \frac{\delta^{2}E}{\delta w_{2}\delta w_{n}}\\ \cdots & \cdots & \cdots & \cdots \\ \frac{\delta^{2}E}{\delta w_{n}\delta w_{1}} & \frac{\delta^{2}E}{\delta w_{n}\delta w_{2}} & \cdots & \frac{\delta^{2}E}{\delta w_{n}^{2}} \end{array}\right],\\ g=\frac{\delta E\left(x,w\right)}{\delta w}={\left[\begin{array}{cccc} \frac{\delta E}{\delta w_{1}} & \frac{\delta E}{\delta w_{2}} & \cdots & \frac{\delta E}{\delta w_{n}} \end{array}\right]}^{T},\\ E\left(x,w\right)=\frac{1}{2}\sum_{p=1}^{P}\;\sum_{m=1}^{M}{\left(d_{pm}-o_{pm}\right)}^{2} \end{array}$$

*x* is the input (vector) applied, *d*_*pm*_ is *m*th output of the network when *p*th input vector is applied, and *o*_*pm*_ is the desired mth output of the network for the *p*th input vector. 

For designing a network, a set of experimental data were taken as examples. Out of the available data of 255 subjects, 75% data were randomly selected for training the network and 25% were used for testing the trained model. Matlab 7.8 (MathWork, Inc., USA) was used to model the current study.

**Figure 2:**
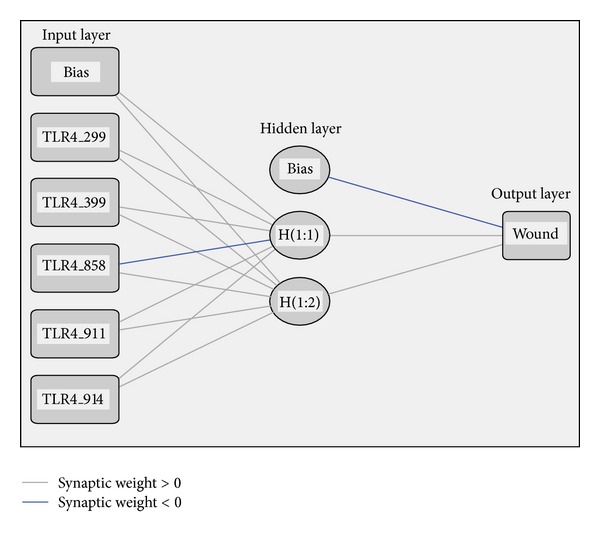
The architecture of the multilayered feedforward artificial neural network (ANN) model for predicting individual risk of development of DFU in T2DM cases.

All the above five SNPs were selected as inputs and output layer consists of one output neurons representing the risk of DFU in T2DM patients. The numbers of hidden layer neurons were decided by training and predicting the “training data” and “testing data” by varying the number of neurons in the hidden layer. A suitable configuration of those neurons had to be chosen. Although different configurations are possible, a configuration having 10 neurons in the hidden layer was chosen. Thus, the final architecture was selected consisting of 5 input nodes, 10 hidden layer nodes, and one output node.  Figure 2 represents the architecture of the ANN model of the study. A test set was used to evaluate the predicting capabilities of the network after each epoch. The weights and biases were stored whenever the error in the predictions reached a minimum.

### 2.6. Multivariate Regression (MLR) Modeling

Conventional statistical multivariate regression was carried out using the same set of data which were used for training and testing the neural networks model. The results were used for validating the prediction results of artificial neural networks model.

For carrying out multivariate linear regression (MLR), statistical software package SPSS [18] was used. The SNPs and the risk of DFU were selected as independent variables, and dependent variables, respectively. The analysis resulted in the equation of the general form *y*′ = *c* + *b*_1_ *x*_1_ + *b*_2_ *x*_2_ + ⋯+*b*_*n*_ *x*_*n*_, where *y*′ is the dependent variable, *c* is a constant, *x*_1_ to *x*_*n*_ are variables and *b*_1_ to *b*_*n*_ are partial regression coefficients for *x*_1_ to *x*_*n*_. The actual prediction equation obtained is as follows:

 risk of wound development = 0.19 + 0.301 (rs4986790) + 0.655 (rs4986791) + 0.17 (rs11536858) + 0.25 (rs1927911) + 0.096 (rs1927914).

## 3. Results

### 3.1. Case—Control Study

In the present study the genotype and haplotype frequencies of the SNPs rs4986790, rs4986791, rs11536858, rs1927911, and rs1927914 were analyzed and the correlation between the carrier status of polymorphisms and susceptibility to develope DFU was also evaluated. Genotype frequency of TLR4 SNP rs4986790, rs4986791, rs11536858, rs1927911, and rs1927914 is summarized in Table 3. We observed that both allelic and the genotypic frequencies of all SNPs were in Hardy Weinberg equilibrium in both study groups.

 The frequency of risk genotype GG of rs4986790 was 0% in controls while it was 0.8% in DFU cases (Table 3). The frequency of the combined risk genotypes GG + AG of rs4986790 was significantly higher in the DFU cases (33.6%) as compared to that in the controls (22.3%) (OR of 1.75 and 95% CI of 1.01 to 3.02). The mutant Genotype TT of rs4986791 was significantly higher in DFU (4.8%) with respect to controls (1.6%) (OR of 4.1, 95% CI of 1.0 to 17.2, *P* value = 0.05). The combined risk genotype CT + TT of TLR4 SNP rs4986791 polymorphism was also significantly higher in DFU cases (40.8%) than in controls (16.2%) (OR of 3.35 and 95% CI of 1.9 to 5.8, *P* value of <0.0001). For TLR4 SNP rs11536858 both the risk genotype GG and combined risk genotypes AG + GG were significantly associated with DFU with respect to controls (OR of 3.8, 95% CI of 1.2 to 12.7, *P* value = 0.02 for GG and OR of 1.7, 95% CI of 1.06 to 2.86, *P* value = 0.02). For TLR4 rs1927914 only the risk genotype CC was significantly associated with DFU compared to controls (OR of 5.6, 95% CI of 1.8 to 17.4, *P* value = 0.003). For rs1927914 the frequency of risk genotype TT was slightly but nonsignificantly higher in DFU (15.2%) compared to controls (11.5%) (OR = 2.0, 95% CI = 0.9 to 4.3) but the combined risk genotype CT + TT was significantly associated with DFU compared to controls (OR = 2.0, 95% CI = 1.2 to 3.2, *P* value = 0.007).

**Figure 3:**
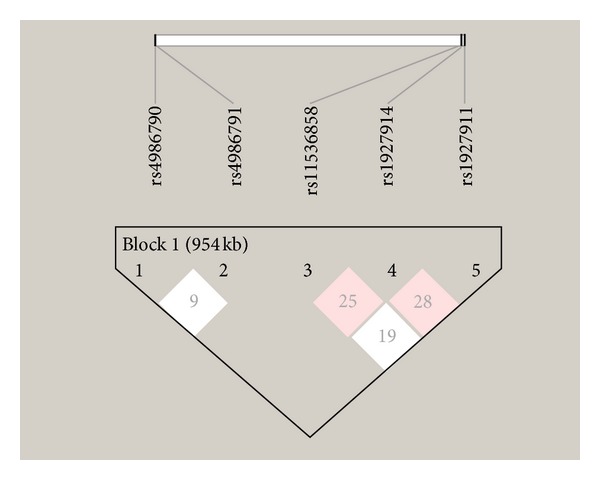
Linkage disequilibrium plot: the LD parameter *D* is represented by the specific value in each cell. The cells are color graduated representing strength of LD between the two markers. The rs numbers are SNP IDs extracted from Ensembl database. The loci rs11536858, rs1927911, and rs1927914 are in intermediate LD.

The SNPs rs4986790, rs4986791, rs11536858, rs1927911, and rs1927914 were selected for the LD and haplotype analysis (Table 4, Figure 3). The SNPs rs4986790 and rs4986791 were not in significant LD (*D*′ = 0.09, LOD = 0.01, confidence bound = 0.01 to 0.74, *r* ^2^ = 0.0). The loci rs11536858 and rs1927914 showed intermediate evidence of LD (*D*′ = 0.25, LOD = 2.34, confidence bound = 0.12 to 0.37, *r* ^2^ = 0.062) so did the loci rs1927911 and rs1927914 (*D*′ = 0.29, LOD = 2.1, confidence bound = 0.13 to 0.42, *r* ^2^ = 0.054). The other two loci rs11536858 and rs1927911 did not show any significant LD (*D*′ = 0.194, LOD = 1.17, confidence bound = 0.05 to 0.33, *r* ^2^ = 0.027). A total of fifteen haplotypes having frequency of more than 1% was found (Table 5). 

### 3.2. Predictive Performance

**Figure 4:**
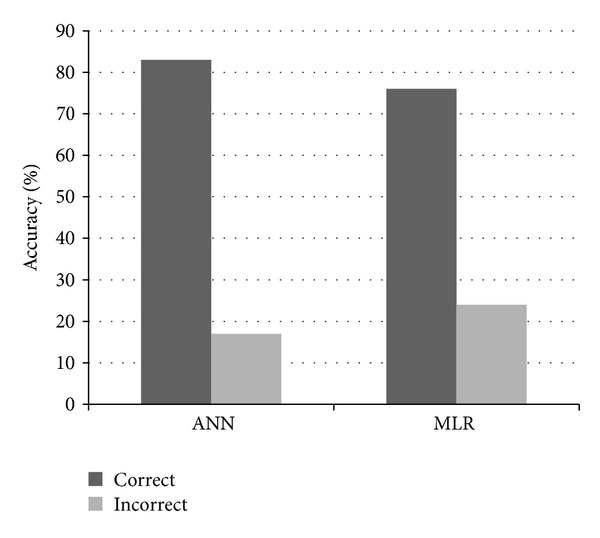
Bar graph showing correct and incorrect prediction in case of ANN model and MLR model.

The trained network was used to validate a set of testing data. The ANN model with the five SNPs as inputs was able to predict 83% of the validation set correctly and 17% of the data incorrectly (Figure 4). The conventional statistical model MLR could predict 74% of the cases correctly in the same validation set of data.

## 4. Discussion

Through various animal models and human studies it has now been established that persistent hyperglycemia in T2DM elicits the innate immune system and chronic low grade inflammation [19]. Polymorphism in inflammatory and immune response genes are mainly related to the alteration in protein functioning that may hamper in the recognition of bacteria by the immune system and in the variation in the level of cytokine response [20]. Genetic susceptibility to secondary complication of T2DM like DFU is multifactorial and risk may also involve factors that are related to the activation of the immune system, and hence, SNPs of series of low penetrance alleles may play an important role in DFU susceptibility [21]. TLRs are a family of evolutionarily highly conserved transmembrane proteins mainly expressed on the surface of immune cells and serve as pattern recognition receptors in mammals [22]. They play a pivotal role in immune responses by regulating inflammatory reactions and activating adaptive immune response to eliminate infectious pathogens and cellular debris [23–25]. There are 10 TLRs expressed in human beings [26]. Once activated TLRs interact with various adapter proteins that control a series of steps leading to expression of genes involved in suppression of inflammatory processes. TLR4 is the first TLR explored in mammals and in addition to serving as a receptor of LPS derived from Gram negative bacteria, it also binds with other exogenous and endogenous ligands like low density lipoprotein, Hsp 60, Hsp 70, fibrinogen, and fibronectin [27–29]. These ligands are also found to be elevated in diabetic patients [30–32]. Recently Chen et al. (2013) have shown TLR4 as an important regulator of early wound healing using TLR4-deficent (C3H/HeJ) mice model [10]. Another two recent studies by Ruzehaji et al. (2013) and Kanhaiya et al. (2013) have clearly shown that any deregulation in the TLR4 mediated downstream signaling may lead to chronic nonhealing ulcers in murine model and humans, respectively [11, 12].

The present study consisted of a total of 5 SNPs in TLR4 gene out of which two are nonsynonymous SNPs Asp299Gly (A > G) and Thr299Ile (C > T) located in the third exon of TLR4 gene situated over the extracellular domain of TLR4. They interfere in the adequate expression and functioning of TLR4, and hence, they have been widely studied in several diseases. TLR4 Asp299Gly polymorphism has been shown to be associated with inflammatory diseases like Crohn's disease [33] and gastric cancer [34] and gastric lymphoma in different cohorts. TLR4 Thr399Ile polymorphism has been established as a genetic risk for gastritis and precancerous lesions in a north Indian population instead of Asp299Gly polymorphism by Achyut et al. [35]. Various epidemiological studies also suggest that these SNPs in the innate immune system may influence the risk of patients to serious infection [36]. Budulac et al. (2011) have shown the association of TLR4 polymorphisms with infection status and disease outcome in Chronic Obstructive Pulmonary Disease while Miedema et al. (2011) showed that TLR4 polymorphisms were associated with risk of developing neutropenia in leukemic children [37, 38]. The remaining three SNPs were rs11536858, rs1927911, and rs1927914 of TLR4 gene and have been shown to be associated with several inflammatory diseases including cancer [10]. 

The data regarding the role of TLR4 gene polymorphism in diabetic complication is sparse. Rudofsky et al. in 2004 [39] showed that Asp299Gly and Thr399Ile genotypes of the TLR4 gene were associated with reduced prevalence of diabetic neuropathy in type 2 diabetes. To the best of our knowledge so far there is no study available till date on association of analyzed SNPs of TLR4 gene and DFU risk. In the present study we found a significant association of these SNPs in the pathogenesis and progression of DFU. The predicted haplotypes of these SNPs suggested a strong evidence of recombination. Out of 15 haplotypes, ACATC (*P* value = 9.3*E* − 5) was significantly associated with the risk of development of DFU in T2DM patients while two haplotypes ATATC (*P* value = 0.012) and ATGTT (*P* value = 0.008) were found to be protective against DFU in a north Indian population (Table 5). The rest other haplotypes were distributed nonsignificantly between the DFU and controls.

Multiple regression analysis and ANN modeling were done in order to model the risk of development of wound in T2DM patients compared to controls. It was found that the predictive ability of ANN model is better than the statistical MLR model and can be used as a predictive tool for the risk assessment of DFU in T2DM patients with further study and training of the model.

In conclusion risk genotypes of SNPs rs4986790, rs4986791, rs11536858, rs1927911, and rs1927914 in the TLR4 gene individually or in combination may impair the wound healing process in T2DM patients resulting in nonhealing DFU. 

## Figures and Tables

**Table 1:** Biochemical and demographic parameters of DFU patients (*N* = 125). Data are presented as mean ± SD or as number (percentage).

Parameters	Values
Average age	56.38 ± 8.62 years
Average BMI (kg/m^2^)	22.19 ± 2.62 Kg/m^2^
Average duration of type 2 diabetes in years	9.25 ± 4.7 years
Male	84 (67.2%)
Female	41 (32.8%)
Poor glycemic control (FBS > 140 mg/dL, PPBS > 180 mg/dL)	73 (58.2%)
Family history present	19 (15.2%)
Nephropathy present (serum creatinine > 1.4 mg/dL)	37 (30.83%)
Neuropathy present (by monofilament test)	73 (58.4%)
Hypertension present (systolic BP > 140 mm of Hg)	44 (35.20%)
Retinopathy present	16 (12.8%)
Dyslipidemia present (serum cholesterol and Tgy levels > 200 mg/dL)	22 (17.6%)
Infection present (wound culture positive for microbes)	67 (53.6%)
Bone involvement (osteomyelitis)	42 (33.6%)

**Table 2:** Primers for PCR-RFLP of the TLR4, restriction enzymes used, and base pair products for genotypes.

SNP ID	Forward primer	Reverse primer	Restriction enzyme used	Product size and genotypes
rs4986790	CTGCTCTAGAGGGCCTGTG	TTCAATAGTCACACTCACCAG	*BccI *	140 = AA 140, 77, 63 = AG 77, 63 = GG
rs4986791	CTACCAAGCCTTGAGTTTCTG	AAGCTCAGATCTAAATACT	*BslI *	110 = TT 110, 89, 22 = TC 89, 22 = CC
rs11536858	ATAACCTCAGTGGGCTCTGG	ATGTTCTGGCATCTGGGAAG	*KpnI *	241 = AA 241, 190, 51 = AG 190, 51 = GG
rs1927911	TCACTTTGCTCAAGGGTCAA	AAACCTGCATGCTCTGCAC	*StyI *	203 = TT 203, 178, 25 = TC 178, 25 = CC
rs1927914	ACAAAATGGTCCCTCACAGC	TGGAAAGTAGCAAGTGCAATG	*SphI *	150 = TT 157, 90, 67 = TC 90, 67 = CC

**Table 3:** Genotype frequencies of single nucleotide polymorphisms (SNPs) rs4986790, rs4986791, rs11536858, rs1927911, and rs1927914 of TLR4 gene among Diabetic Foot Ulcer (DFU) and controls. The differences in frequencies between the DFU and control groups were analyzed for statistical significance at the 95% confidence interval using chi square test. Odds ratios (ORs) were calculated and reported within the 95% confidence limits. A two-tailed *P* value of ≤0.05 was considered as statistically significant.

SNP and genotype	Controls, number, (%)	Cases, number, (%)	OR	95% CI	*P* value
rs4986790 (TLR4_Asp299Gly) 119515123					
AA	101 (77.7)	83 (66.4)	—	—	—
AG	29 (22.3)	41 (32.8)	1.7	1.0 to 3.0	0.05
GG	00 (00)	1 (0.8)			
AG + GG	29 (22.3)	42 (33.6)	1.75	1.01 to 3.02	0.04

rs4986791 (TLR4_Thr399Ile) 119515423					
CC	109 (83.8)	74 (59.2)	—	—	—
CT	19 (14.6)	45 (36.0)	3.3	1.9 to 5.8	0.00003
TT	2 (1.6)	6 (4.8)	4.1	1.0 to 17.2	0.05
CT + TT	21 (16.2)	51 (40.8)	3.35	1.9 to 5.8	0.000003

rs11536858 (TLR4_1859) 120464147					
AA	68 (52.3)	48 (38.4)	—	—	—
AG	59 (45.3)	68 (54.4)	1.6	1.0 to 2.7	0.05
GG	3 (2.4)	9 (7.2)	3.8	1.2 to 12.7	0.02
AG + GG	62 (47.7)	77 (61.6)	1.7	1.06 to 2.86	0.02

rs1927914 (TLR4_2437) 120464725					
TT	65 (50)	63 (50.4)	—	—	—
TC	64 (49.2)	50 (40.0)	0.8	0.5 to 1.3	0.40
CC	1 (0.8)	12 (9.6)	5.6	1.8 to 17.4	0.003
TC + CC	65 (50)	62 (49.6)	0.98	0.6 to 1.6	0.94

rs1927911 (TLR4_7764) 120470054					
CC	63 (48.5)	40 (32.0)	—	—	—
CT	52 (40)	66 (52.8)	2.0	1.2 to 3.3	0.011
TT	15 (11.5)	19 (15.2)	2.0	0.9 to 4.3	0.08
CT + TT	67 (51.5)	85 (68.0)	2.0	1.2 to 3.2	0.007

**Table 4:** The table describes the LD value calculated for all the present SNPs of TLR4 gene. L1 and L2 are loci in question, *D*′ is the value of *D* prime between the two loci, LOD is the log of the likelihood odds ratio, *r* ^2^ is the correlation coefficient between the two loci, CI low is 95% confidence lower bound on *D*′, CI  high is the 95% confidence upper bound on *D*′, Dist is the distance (in bases) between the loci and is only displayed if a marker info file has been loaded, and *T*-int is a statistic used by the HapMap Project to measure the completeness of information represented by a set of markers in a region.

L1	L2	*D*	LOD	*R* ^2^	CI low	CI high	Dis	*T*-int
rs4986790	rs4986791	0.09	0.01	0.0	0.01	0.74	300	0.01
rs11536858	rs1927914	0.25	2.34	0.062	0.12	0.37	578	3.51
rs11536858	rs1927911	0.19	1.17	0.027	0.05	0.33	5907	—
rs1927914	rs1927911	0.29	2.1	0.054	0.13	0.42	5329	3.27

**Table 5:** Association status of common haplotypes of TLR4 gene with Diabetic Foot Ulcers. Fifteen haplotypes with a frequency of more than 1% was found out of which one haplotype ACATC (*P* value = 9.3*E* − 5) was found to be associated with DFU while two haplotypes ATATC (*P* value = 0.012) and ATGTT (*P* value = 0.008) were found to be protective against DFU in a north Indian population.

Haplotype	Frequency	Case, control frequencies	Chi square	*P* value
ACATC	0.280	0.355, 0.204	15.278	9.2789*E* − 5
ACATT	0.136	0.140, 0.130	0.115	0.7351
ACGTC	0.097	0.109, 0.085	0.83	0.3623
ACACC	0.082	0.086, 0.078	0.131	0.7174
ACGCT	0.077	0.065, 0.090	1.202	0.273
ATATC	0.069	0.042, 0.097	6.33	0.0119
GCATC	0.050	0.053, 0.048	0.067	0.7951
GCGTT	0.027	0.020, 0.034	1.013	0.3143
ATGCC	0.025	0.017, 0.033	1.566	0.2108
ACACT	0.024	0.033, 0.015	1.809	0.1786
ATACT	0.022	0.014, 0.030	1.679	0.1951
ACGTT	0.019	0.010, 0.027	2.166	0.1411
ATGTT	0.018	0.003, 0.034	6.88	0.0087
GCATT	0.015	0.008, 0.022	1.961	0.1614
GCACT	0.013	0.012, 0.014	0.06	0.8067
